# Niraparib-induced STAT3 inhibition increases its antitumor effects

**DOI:** 10.3389/fonc.2022.966492

**Published:** 2022-10-17

**Authors:** Qianqian Zhao, Adrian Kohut, Yi-Jia Li, Antons Martincuks, Theresa Austria, Chunyan Zhang, Nicole Lugo Santiago, Rosemarie Martinez Borrero, Xuan Thuy Phan, Laleh Melstrom, Lorna Rodriguez-Rodriguez, Hua Yu

**Affiliations:** ^1^ Department of Immuno-Oncology, Beckman Research Institute, City of Hope National Medical Center, Duarte, CA, United States; ^2^ Irell and Manella Graduate School of Biological Sciences, City of Hope National Medical Center, Duarte, CA, United States; ^3^ Department of Surgery, Division of Gynecologic Oncology, City of Hope National Medical Center, Duarte, CA, United States; ^4^ Department of Surgery, Division of Surgical Oncology, City of Hope National Medical Center, Duarte, CA, United States

**Keywords:** niraparib, STAT3, SRC, pancreatic cancer, ovarian cancer

## Abstract

Recently, poly(ADP-ribosyl)ation polymerase inhibitors (PARPis), which induce synthetic lethality of tumor cells with DNA damage repair defects, have emerged as a promising therapy for ovarian, breast, and pancreatic cancer. Although the PARPi Olaparib is limited to treating cancer patients with DNA repair deficiencies, the PARPi Niraparib is FDA approved to treat ovarian cancer patients regardless of their status in DNA repair pathways. Despite differences in the affinity to PARP enzymes, the rationale behind the clinical use of Niraparib in patients without DNA repair deficiencies is still lacking. Moreover, only Olaparib has been approved for pancreatic ductal adenocarcinoma (PDAC) patients with BRCA mutations, accounting for only 5-7% of total PDACs. It remains unclear whether Niraparib could be beneficial to PDACs without BRCA mutations. We found that Niraparib inhibits ovarian and PDAC tumor cell growth, regardless of BRCA mutational status, more effectively than Olaparib. Unlike Olaparib, which is known to activate STAT3, Niraparib inhibits STAT3 activity in ovarian and PDAC cancer cell lines and patient tumors. Moreover, Niraparib regulates the expression of several STAT3 downstream genes involved in apoptosis. Overexpression of a constitutively activated STAT3 mutant rescues Niraparib-induced cancer cell apoptosis. Our results suggest that Niraparib inhibits pSTAT3 by interfering with SRC tyrosine kinase. Collectively, our studies provide a mechanism underlying Niraparib’s ability to induce tumor cell apoptosis without BRCA mutations, suggesting the potential use of Niraparib for treating PDAC patients regardless of BRCA status.

## Introduction

Ovarian cancer is the most lethal gynecologic malignancy and the fifth leading cause of cancer-related mortality among women in the United States. A combination of cytoreductive surgery plus platinum and taxane-based chemotherapy has typically allowed for high initial remission rates; however, most patients experience tumor recurrence and succumb to the disease. Fortunately, treatment paradigms have been revolutionized by incorporating poly(ADP-ribose) polymerase inhibitors (PARPis) into the management of ovarian cancer. Recently, PARPis, which target the key enzyme PARP in DNA damage response, have demonstrated potent antitumor effects due to synthetic lethality exerted on tumor cells deficient in DNA damage repair, most remarkably those with BRCA1/2 mutations (1, 2). Three PARPis, including Olaparib, Rucaparib, and Niraparib, are currently FDA approved for the management of BRCA mutated or homologous recombination deficient (HRD) ovarian cancers. However, only Niraparib has demonstrated clinical efficacy and gained FDA approval for use in non-BRCA mutated and homologous recombination proficient ovarian cancers (3).

Despite promising antitumor efficacy, PARPis still show limitations in the clinic, as resistance to PARPis occurs in most treated patients (4). It has been demonstrated that the lack of long-term antitumor efficacy of PARP inhibitor, Olaparib, is partially attributed to its activation of Signal Transducer and Activator of Transcription 3 (STAT3) in tumors and the tumor microenvironment (5, 6). STAT3, as a signal transducer and transcription factor, plays an essential role in cancer malignancy. The binding of growth factors and cytokines such as EGF and IL6 to cell surface receptors activates the STAT3 pathway, leading to STAT3 Y705 phosphorylation and dimerization, followed by nucleus translocation and gene regulation (7). Aberrant STAT3 activation in tumors contributes to tumor cell proliferation/survival, metastasis, and therapy resistance (8–10). Moreover, elevated STAT3 activity in tumor-associated immune cells leads to an immunosuppressive microenvironment, further promoting tumor progression. Conversely, blocking STAT3 significantly inhibits tumor growth by directly killing tumor cells and boosting antitumor immune responses (11).

In addition to ovarian cancers, Olaparib has been approved to treat pancreatic ductal adenocarcinoma (PDAC) patients with germline BRCA1/2 mutations (12). However, only 5-7% of the patients carry germline BRCA mutations, leaving most patients without the benefits of PARPis (13, 14). Moreover, only Olaparib has FDA approval for PDAC maintenance therapy (12). In ovarian cancer, Niraparib is FDA-approved to treat patients regardless of BRCA status (15); however, whether Niraparib would effectively kill PDAC tumor cells without BRCA mutations is unknown.

Although Niraparib is prescribed to ovarian cancer patients irrespective of BRCA status, the scientific rationales and mechanism(s) underlying such use remain to be further investigated. Nevertheless, previous studies have demonstrated that Niraparib provides more effective cytotoxicity on tumor cells than Olaparib (16, 17). In addition, the two PARP inhibitors possess distinct biophysical characteristics (18), which provides an explanation that, as PARP inhibitors in targeting DNA damage/repair deficiency, Niraparib is more potent in killing tumor cells than Olaparib. However, the mechanism(s) by which Niraparib is more effective than Olaparib in killing BRCA proficient tumor cells remains unexplained. The reduced antitumor effects of Olaparib in ovarian cancer cells can be attributed partially to the induction of STAT3 phosphorylation through decreasing PolyADP-ribosylation (PARylation). As a PARP inhibitor, Niraparib also inhibits PARylation (19), which is predicted to increase phosphorylated STAT3 (pSTAT3). Unexpectedly, we discovered that, rather than increasing pSTAT3, Niraparib inhibits pSTAT3. To investigate the possibility that the ability of Niraparib to inhibit pSTAT3 provides additional antitumor effects regardless of BRCA mutation status, we included various PDAC and ovarian cancer cell lines carrying either wild-type or mutated BRCA genes. We also determined whether Niraparib’s superior antitumor effects are contributed by its inhibitory effects on pSTAT3. We further sought to determine how Niraparib inhibits pSTAT3 in cancer cells. Our findings provide mechanistic insight into the differential antitumor activities between Olaparib and Niraparib, which may improve the clinical use of these two drugs and promote Niraparib for treating PDAC with or without BRCA mutations.

## Materials and methods

### Patients and tumor samples

The human tumor specimens from ovarian cancer and pancreatic cancer patients were obtained through IRB-approved protocols (#18004, #19450, and #06129) at City of Hope. The pancreatic cancer patient-derived xenografted (PDX) tumor (UPN40) was a generous gift from Dr. Jianhua Yu at City of Hope. The paraffin-embedded human PDAC tumor tissue slides were kind gifts from Dr. Haiyong Han at TGen.

Tumor and immune cells from ovarian patient ascites were obtained through centrifugation. Red blood cells were removed, and the remaining cells were resuspended in culture medium and seeded at 1x10^6^ cells per well in 24-well plates and subsequently treated for 24 hours with the indicated concentrations of Niraparib shown in the figures.

The single-cell suspensions of patient or PDX tumors were prepared as previously described (20). Tumor cells were seeded in each well (1x10^6^ cells/well) of 24-well plates and treated for 24 hours with Niraparib or DMSO, as indicated in each figure.

### Antibodies and reagents

The primary antibodies and reagents used are summarized in **Supplementary Table 2**.

### Cell culture

MIA PaCa-2, PANC-1, Capan-1, and OVCAR8 cells were obtained from ATCC. Mouse KPB cells were a generous gift from Dr. Xiaochun Yu (21). PEO1 ovarian cancer cells were purchased from Sigma (#10032308). Mouse KPC cells were a generous gift from Dr. Laleh Melstrom. Wild-type and STAT3C-overexpressing mouse fibroblast cells (3T3) were a generous gift from Dr. James E. Darnell. All cells were cultured in DMEM supplemented with 10% FBS, 0.2% MycoZap™ Plus-CL (#VZA-2012, Lonza), and 1x Antibiotic-Antimycotic (#15240-062, Gibco), and grown at 37°C in a 5% CO_2_ humidified incubator.

### Cell viability assay

Cells were seeded at 3,000 cells per well in 96-well plates. The next day, cells were treated for 48 hours with DMSO or different concentrations of Olaparib or Niraparib as indicated. Cell viability was measured by CellTiter-Glo^®^ Luminescent Cell Viability Assay (#G7570, Promega) according to the manufacturer’s protocol. Luminescence was detected on a Cytation 5 Cell Imaging Multi-Mode Reader (BioTek). Three independent experiments were conducted in at least triplicate.

### Colony formation assay

Cells were plated at 3,000 cells per well in a 6-well plate and the next day were treated with DMSO, 10 µM Olaparib, or 10 µM Niraparib. After 7-10 days, cells were fixed and stained with 0.5% w/v crystal violet solution in 25% methanol for 30 minutes. Colonies were then counted. Each experiment was performed three times.

### Real-time PCR

Total RNAs were extracted from cells using a RNeasy Mini Kit (QIAGEN) according to the manufacturer’s protocol. RNA (0.5-1 µg) was reverse-transcribed to cDNA using an iScript™ cDNA Synthesis Kit (Bio-Rad). Real-time PCR reactions were conducted as previously described, and samples were run in triplicate (22). The 18S rRNA was used as an internal control to normalize mRNA levels.

Primer Sequences: hBCL2L1 F: 5’-GTCCTCACTCCCAGTCCAA-3’, R: 5’-GCTGAGGCCATAAACAGCC-3’; hCASP3 F: 5’-ACATGGCGTGTCATAAAATACC-3’, R: 5’-CACAAAGCGACTGGATGAAC-3’; hCASP8 F: 5’-ATGCAAACTGGATGATGACA-3’ R: 5’-GATTATCTTCAGCAGGCTCTT-3’; hCASP9 F: 5’-TGTCCTACTCTACTTTCCCCAGGT TTT-3’, R: 5’-GTGAGCCCACTGCTCAAAGAT-3’; h18S F: GTAACCCGTTGAACCCCATT, R: GGACATCTAAGGGCATCACA.

### Western blot

Cells were lysed in SDS buffer (100mM Tris-Cl pH6.8, 4% w/v SDS, 20% v/v glycerol, 0.2% w/v bromophenol blue), and protein concentration was measured using a Pierce™ BCA Protein Assay Kit (#23228, ThermoFisher Scientific). Proteins (30 μg/lane) were subjected to SDS-PAGE and then transferred to a PVDF membrane (#10600023, Cytiva) for Western blotting. Primary and secondary antibodies used for immunoblotting are listed in **Supplementary Table 2**. Protein bands were visualized with a Chemiluminescent Detection Kit (#34096, ThermoFisher Scientific).

### Apoptosis analysis

Cells were treated as indicated in the figure legend and then stained with 5 µl Annexin V-FITC or 5 µl Annexin V-APC and 5 µl propidium iodide in 100 µl binding buffer (#550475, #556547, BD Pharmingen) for 15 min at room temperature (RT, 25°C) in the dark. Cells were then added to a 400 µl binding buffer and analyzed by flow cytometry (BD LSRFortessa). Data were assessed using FlowJo software (RRID: SCR_008520).

### Transfection

OVCAR8 cells were seeded at 3x10^5^ cells per well in a 6 well-plate and transfected with Stat3-C Flag pRc/CMV plasmid (23) or pRc/CMV plasmid (V75020, Invitrogen) using lipofectamine 2000 (#11668027, Invitrogen) according to manufacturer’s protocol. The next day, cells were treated with 1 mg/ml Geneticin (#10131027, ThermoFisher Scientific) and subjected to single colony selection. STAT3 expression was analyzed by Western blot. The Stat3-C Flag pRc/CMV plasmid was a gift from Jim Darnell (RRID: Addgene_8722; http://n2t.net/addgene:8722).

To establish STAT3C-overexpressing cells, a lentivirus carrying STAT3C with a GFP reporter gene was used to transduce MIA PaCa-2 and PANC-1 cells. After 24-hour transduction, MIA PaCa-2 cells were subjected to a selection of GFP-positive cells on a FACSAria™ III Cell Sorter (BD). STAT3 expression of GFP-positive cells was analyzed by Western blot. The EF.STAT3C.Ubc.GFP plasmid was a gift from Linzhao Cheng (RRID: Addgene_24983; http://n2t.net/addgene:24983). The FUGW plasmid was a gift from David Baltimore (RRID: Addgene_14883; http://n2t.net/addgene:14883).

MIA PaCa-2 cells were transfected with PARP1 siRNA (6) or control siRNA (sc-44236, Santa Cruz Biotechnology) for 48 hours using Lipofectamine RNAiMAX Transfection Reagent (#13778, ThermoFisher Scientific) according to manufacturer’s protocol.

### In-cell thermal shift assay

An in-cell thermal shift assay was performed as previously described (24). In brief, cells were harvested and resuspended in fresh medium at a density of 0.5 x 10^6^ cells/ml (OVCAR8) or 1 x 10^6^ cells/ml (MIA PaCa-2). Niraparib (20 µM) or DMSO (0.04%) was added to the culture medium, and cells were incubated at 37°Cfor 2 hours. Afterward, cells were resuspended in PBS with protease inhibitors (Roche) and aliquoted into 0.2 ml PCR tubes containing 0.5 x 10^6^ cells. Cells were heated using a DNAEngine Peltier Thermal Cycler (BioRad) at designated temperatures (40-64°C for OVCAR8; 40-55°C for MIA PaCa-2) for 3 min. Immediately after heating, the samples were incubated at RT for 3 min, followed by several freeze-thaw cycles to lyse the cells. Finally, the cell lysates were spun down at 20,000×g for 20 min at 4°C to pellet cell debris. The supernatant was used for Western blot analysis.

### *In vitro* thermal shift assay

*In vitro* thermal shift assay was conducted as described previously (25). In short, human SRC kinase (final 1.14 µM in each reaction, #10755-H20B, Sino Biological) diluted in PBS was mixed thoroughly with SYPRO Orange (final 4x, S6650, Invitrogen) and followed by adding Niraparib or Dasatinib (BMS-354825) at the concentrations listed in the Figure legend. The reactions were performed in triplicate using a CFX96 Real-time PCR Detector (Bio-Rad). Melting curves and melting temperatures (Tm) were assessed with GraphPad Prism 9.

### *In vitro* culture of tumor tissue slices

The *in vitro* culture of tumor tissue slices was based on a method previously reported (26). Briefly, patient tumor tissues were cut into 3-5 mm^2^ pieces with forceps and scissors. The tissues were subsequently placed in the well of a 24-well culture plate with 1.5 ml of culture medium containing Niraparib, as indicated in the Figure legend. After 24 hours of incubation, the tissues were harvested and subjected to OCT-frozen embedding, followed by pathology processing.

### Immunohistochemical/immunofluorescent staining and confocal microscopy

The OCT-embedded frozen tissue slides were dried at RT, fixed in 2% paraformaldehyde for 15 min, and permeabilized in methanol at -20°C for 30 min. Image-iT^®^ FX signal enhancer (I36933, ThermoFisher Scientific) was applied to the slides according to the manufacturer’s protocol; tissues were blocked with 10% FBS in PBS for 1 hour at RT, incubated with pSRC (1:200, RRID : AB_331697) primary antibody overnight at 4°C, and subjected to Opal520 staining (FP1487001KT, PerkinElmer) according to the manufacturer’s instructions. After Opal520 staining, the primary and secondary antibodies were stripped by microwave heating. The tissues were prepared for incubation with pSTAT3 (1:100, RRID: AB_2491009) overnight at 4°C. pSTAT3 was stained with AF546-conjugated secondary antibody (1:1000) for 1 h in the dark at RT. AF647-conjugated Pan-cytokeratin antibody (1:400) and the Hoechst 33342 (1:1000, #H3570, ThermoFisher Scientific) were used to stain cytokeratin and the nucleus, respectively. Fluorescence images were obtained on a Zeiss LSM 700 confocal microscope (Zeiss, Jena, Germany) using a 20x or 40x immersion objective, and images were assessed by Zen software (Zeiss). pSTAT3 and pSRC levels were quantified using ImageJ software (RRID: SCR_003070) and plotted in GraphPad Prism 9 (RRID: SCR_002798).

Paraffin-embedded human PDAC tumor sections were deparaffinized and rehydrated through xylene and serial ethanol. High pH Tris-based antigen unmasking solution (H-3301, Vector Labs) was then used for antigen retrieval according to the manufacturer’s instructions. IHC-IF staining was performed as described above.

## Results

### Niraparib exhibits more potent antitumor effects than olaparib, regardless of tumor BRCA status in PDAC

**Figure 1:**
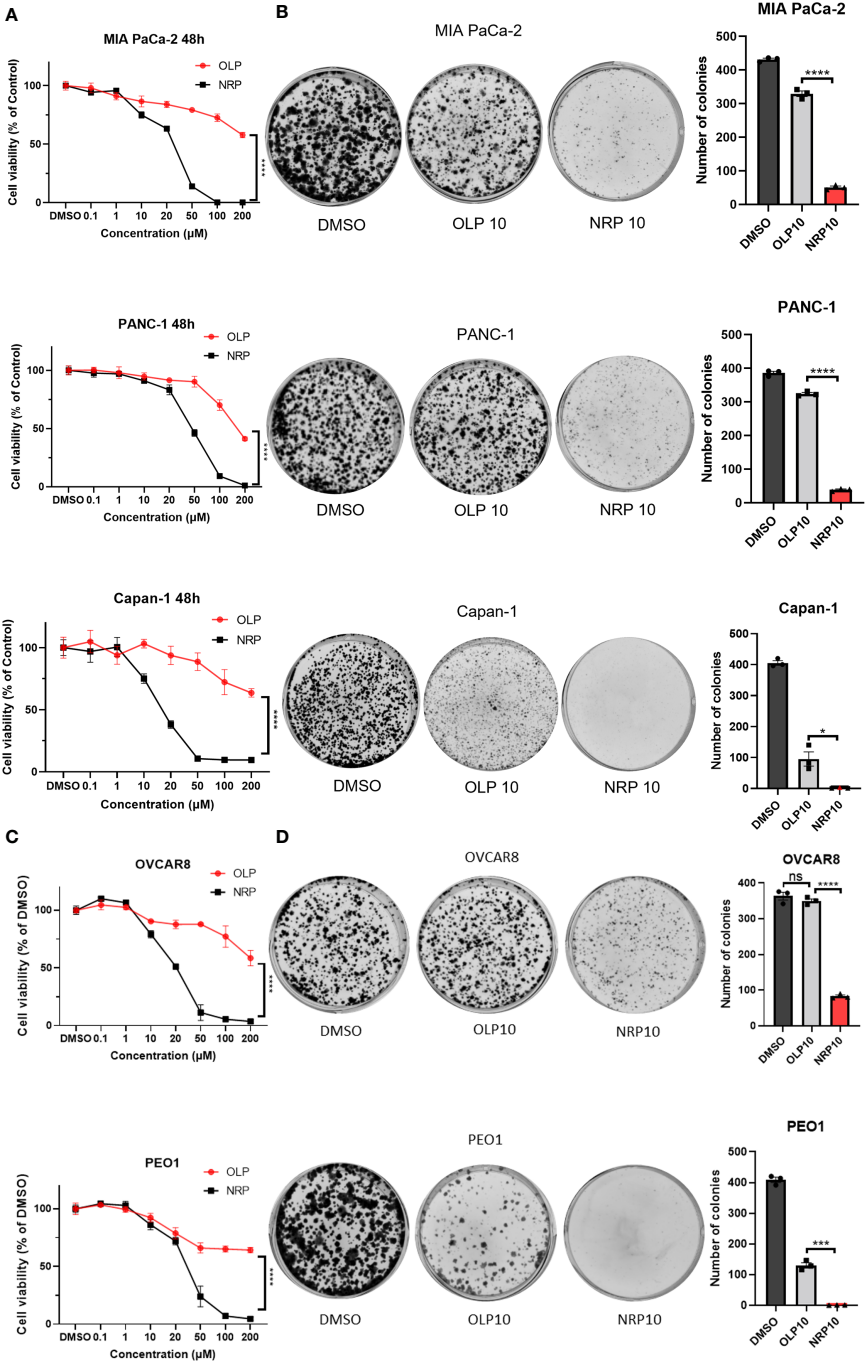
Niraparib (NRP) exhibits more potent antitumor effects than Olaparib (OLP) on PDAC cancer cells with or without BRCA mutations as well as on ovarian cancer (OvCa) cells. **(A)** PDAC cell lines MIA PaCa-2, PANC-1, and Capan-1 were treated with NRP or OLP for 48h. Cell viability was assessed by CellTiter-Glo assay. The data shown are representative of three independent experiments. Two-way ANOVA, ****p<0.0001. **(B)** A colony formation assay was conducted to determine the long-term antitumor effects of NRP and OLP on PDAC cancer cells. The representative images of three independent tests are shown. The number of colonies was counted, and data are shown as mean ± SEM (N=3). Unpaired two-tailed Student t-test, *p<0.05, ****p<0.0001. **(C)** Cell viability assay was conducted on OvCa cell lines OVCAR8 and PEO1 under the same treatment as in **(A)**. Results are representative of three independent experiments. Two-way ANOVA, ****p<0.0001. **(D)** Colony formation assay on OVCAR8 and PEO1 cells. The representative images of three independent experiments are shown here with the quantification of colonies. Data shown are shown as mean ± SEM (N=3). Unpaired two-tailed Student t-test, ns, not significant, ***p<0.0001, ****p<0.0001.

Clinically, Niraparib appears to impede ovarian cancer progression in women with both BRCA wild-type and BRCA mutated tumors, whereas Olaparib primarily significantly affects ovarian tumors with HRD, specifically, BRCA mutated tumors. Therefore, we first sought to determine whether Niraparib exerted antitumor effects on other cancer cells regardless of their DNA damage repair deficiency status and to compare the antitumor cytotoxicity of Niraparib and Olaparib in ovarian cancer and PDAC. To investigate this, we performed cell viability assays on human PDAC and ovarian cancer cells with or without BRCA mutations. The IC50 of Olaparib on BRCA1/2-proficient human pancreatic cancer MIA PaCa-2 and PANC-1 cell lines was 200 µM, while the IC50 of Niraparib was 26 µM and 50 µM, respectively. The IC50 of Olaparib on BRCA2-deficient Capan-1 cells was more than 200 µM, while the IC50 of Niraparib was approximately 15 µM. In addition, Niraparib demonstrated greater potency than Olaparib in inhibiting cancer cell proliferation of both BRCA-proficient and -deficient human pancreatic cancer cells (**Figure 1A**). Cell viability results from KPC BRCA2-proficient murine pancreatic tumor cells and BRCA2-deficient KPB tumor cells confirmed higher cytotoxicity of Niraparib in pancreatic cancer regardless of BRCA status (**Figure S1**). Moreover, the superior antitumor effects of Niraparib compared to Olaparib were also observed in ovarian cancer cells OVCAR8 and PEO1; the IC50 of Olaparib was around 200 µM for both cell lines, and the IC50 of Niraparib was about 20 µM and 28 µM, respectively (**Figure 1C**). OVCAR8 cells are more resistant to PARPi because of their BRCA-proficient phenotype despite heterozygous methylation on the BRCA1 promoter (27, 28), whereas PEO1 cells harbor a truncated BRCA2 mutation representing ovarian cancers deficient in DNA damage repair mechanisms (29). In addition to comparing short-term cytotoxic effects, long-term cell growth inhibition assessed by colony formation assays confirmed that Niraparib was a more effective therapeutic agent *in vitro* than Olaparib in human PDAC cells and ovarian cancer cells with or without BRCA mutations (**Figures 1B, D**). Collectively, these results indicate that the antitumor efficacy of Niraparib is superior to that of Olaparib in PDAC and ovarian cancer cells regardless of BRCA status.

### Niraparib inhibits STAT3 activity

**Figure 2:**
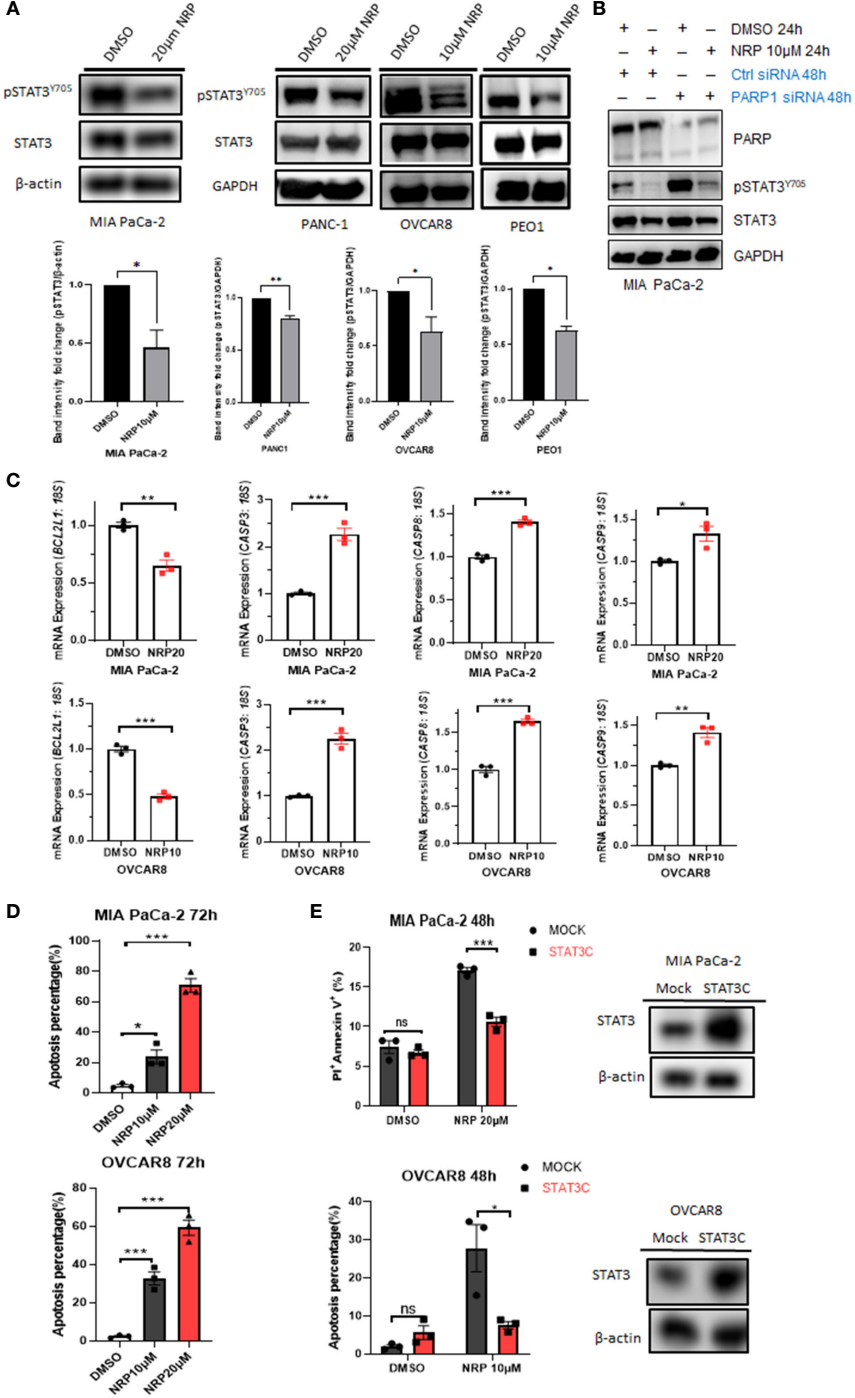
NRP inhibits pSTAT3 and enhances cell apoptosis by regulating STAT3 downstream genes. **(A)** Western blot indicating levels of pSTAT3(Y-705) in MIA PaCa-2, PANC-1, OVCAR8, and PEO1 cells after NRP or DMSO treatment for 24h or 36 hours (PEO1). GAPDH or β-actin served as a loading control. Band intensities from two or three independent experiments were quantified by ImageJ and showed in a bar graph as mean ± SEM **(B)** Western blot indicating the level of pSTAT3(Y-705) in MIA PaCa-2 cells transfected with PARP1 siRNA for 2 days, followed by 24-hour NRP treatment. **(C)** Real-time PCR to examine changes of STAT3 downstream gene expression levels in MIA PaCa-2 and OVCAR8 cells with or without NRP treatment. Gene expression was normalized to the housekeeping gene *18S*. Unpaired two-tailed Student t-test. The data shown are as mean ± SEM (N=3). *p<0.05, **p<0.001, ***p<0.0001. **(D)** NRP-induced apoptosis in MIA PaCa-2 and OVCAR8 cells was examined by Annexin V/PI staining and analyzed by flow cytometry 72h after indicated treatments. Unpaired two-tailed Student t-test. Data are shown as mean ± SEM (N=3). *p<0.05, ***p<0.0001. **(E)** STAT3C overexpression significantly rescued NRP-induced apoptosis. Mock vector or STAT3C-overexpressing MIA PaCa-2 or OVCAR8 cells were treated with NRP for 48h, followed by Annexin V-APC/PI and flow cytometry. Unpaired two-tailed Student t-test. Data are shown as mean ± SEM (N=3). ns, not significant. *p<0.05, ***p<0.0001. Total STAT3 levels in STAT3C-overexpressing cells were examined by Western blot. β-actin served as a loading control.

Recently, our laboratory and others showed that either Olaparib or PARP1 gene silencing activated STAT3 by increasing STAT3 phosphorylation through dePARylation in cancer cells and immune cells, partially counteracting the tumor cell-killing efficacy of Olaparib (5, 6). However, the effect of Niraparib on STAT3 activity had not been studied. Unexpectedly, we found that Niraparib inhibited STAT3 phosphorylation, shown as a reduction in pSTAT3(Y-705) following treatment with Niraparib in both MIA PaCa-2 and PANC-1 human PDAC cell lines and OVCAR8 and PEO1 ovarian cancer cells (**Figure 2A**), despite reduced PARylation by Niraparib (**Figure S2A**). We next examined whether Niraparib inhibited the increased phosphorylation of STAT3 induced by PARP1 gene silencing, which is known to be PARylation-dependent. We observed that Niraparib further abrogated the upregulation of pSTAT3 induced by PARP1 knockdown in MIA PaCa-2 cells, supporting a potent inhibitory effect of Niraparib on pSTAT3 through a non-PARylation mechanism (**Figure 2B**). Moreover, real-time qPCR showed that Niraparib altered the expression of STAT3 downstream target genes, specifically those involved in apoptosis. The anti-apoptotic gene, *BCL-XL* (*BCL2L1*), typically upregulated by STAT3 activation (8), was significantly reduced in MIA PaCa-2 and OVCAR8 cells. Conversely, pro-apoptotic *CASP3*, *CASP8*, and *CASP9* genes, suppressed by STAT3 activity (8, 30), were markedly upregulated (**Figure 2C**).

These gene signatures suggest that Niraparib effectively induces tumor cell apoptosis through STAT3 inactivation. Indeed, flow cytometric analysis of Annexin V staining showed that the percentage of apoptotic cells was dramatically increased in MIA PaCa-2 and OVCAR8 cells after 72-hour incubation with either 10 µM or 20 µM Niraparib (**Figure 2D**). To assess whether Niraparib-induced tumor cell apoptosis is at least partially mediated by its inhibitory effects on STAT3 activity, we tested whether restoring STAT3 activity by ectopic expression of STAT3C, a constitutively active mutant of STAT3 (23), rescued impaired cell viability resulting from Niraparib treatment. We tested this hypothesis using OVCAR8 and MIA PaCa-2 cells expressing ectopic STAT3C, which was confirmed by Western blot (**Figure 2E**). Annexin V staining indicated that ectopic expression of STAT3C significantly reduced the percentage of apoptotic OVCAR8 cells after 48-hour treatment with Niraparib compared to mock control cells (**Figure 2E**).

Additionally, late-stage apoptosis, indicated by the percentage of Annexin V^+^/PI^+^ cells, was dramatically reduced in MIA PaCa-2 cells expressing ectopic STAT3C (**Figure 2E**). Similar rescue from apoptosis was observed in PANC-1 cells expressing ectopic STAT3C (**Figure S2C**). Colony formation assays confirmed the long-term anti-apoptotic effects of STAT3C on tumor cells treated with Niraparib (**Figure S2B**). These data show that restoring STAT3 activity by ectopically expressing STAT3C can rescue the apoptotic phenotype induced by Niraparib. Moreover, in pancreatic and ovarian cancer cells (except for PEO1 cells), total PARylation levels of proteins were significantly reduced when the cells were treated with the same concentration of Niraparib required for pSTAT3 reduction. PEO1 cells required higher concentrations of Niraparib to inhibit PARylation (**Figure S2A**). These data confirm that, in addition to inhibiting PARP catalytic function, the ability of Niraparib to promote cell death in PDAC and ovarian cancer cells regardless of BRCA status is partially mediated by its inhibitory effects on pSTAT3.

### Niraparib inhibits pSTAT3 partially through the downregulation of SRC kinase

**Figure 3:**
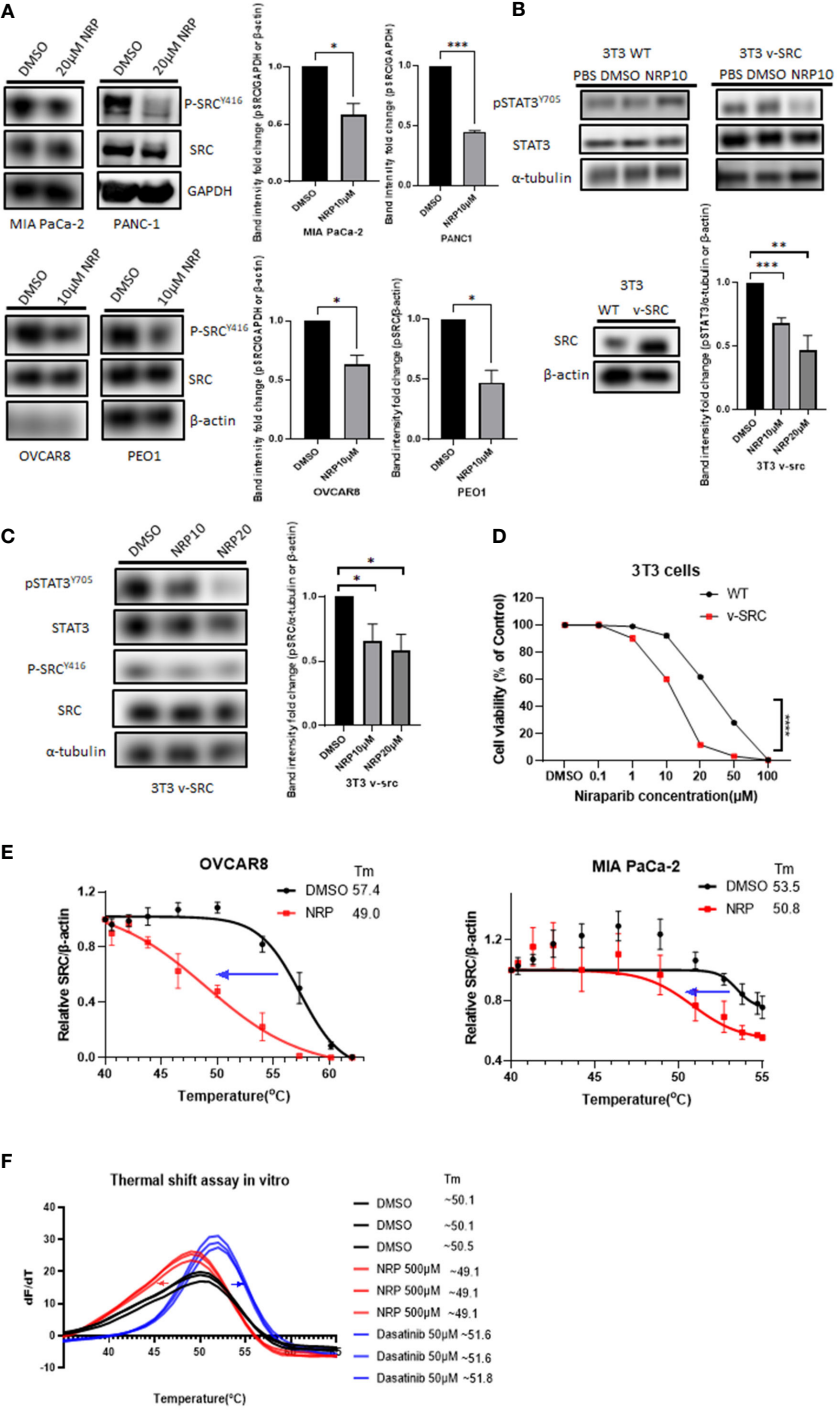
pSRC was downregulated along with reduced pSTAT3 post NRP treatment. **(A)** pSRC in cell lysates described in **Figure 2A** detected by Western blot. GAPDH or β-actin served as the loading control. Band intensities from two or three independent experiments were quantified by ImageJ and showed in a bar graph as mean ± SEM **(B)** 3T3 WT or 3T3 v-SRC cells were treated with DMSO or 10 µM NRP for 24h, and cell lysates were analyzed by Western blot. α-tubulin was detected as the loading control. The right panel shows the protein level of ectopic expression of SRC in 3T3 v-SRC cells by Western Blot. β-actin served as the loading control. Band intensities from three independent experiments were quantified by ImageJ and showed in a bar graph as mean ± SEM **(C)** The level of pSRC was examined in 3T3 v-SRC cells treated with 10 µM or 20 µM NRP for 24h by Western blot. α-tubulin was used as the loading control. Band intensities from three independent experiments were quantified by ImageJ and showed in a bar graph as mean ± SEM **(D)** Cell viability of 3T3 v-SRC or wild-type 3T3 cells after NRP treatment was measured by CellTiter-Glo assay. A representative graph of three independent experiments is shown. **(E)** Thermal stability of endogenous SRC in MIA PaCa-2 or OVCAR8 cells was measured by in-cell thermal shift assay as described in Materials and Methods. Data are shown as mean ± SEM (N=3). **(F)** *In vitro* thermal shift assay was performed in triplicate using human recombinant SRC protein in the presence of DMSO control, NRP, or Dasatinib (positive control). For each condition, all three SRC protein melting curves are shown. *p<0.05, **p<0.001, ***p<0.0001.

To determine how Niraparib could inhibit pSTAT3, we looked into whether it can inhibit the phosphorylation of kinases upstream of STAT3. Both ovarian cancer and PDAC tumors have elevated activity of SRC protein kinase, which is critical for the phosphorylation of STAT3 at tyrosine 705 (31, 32). Therefore, we wanted to test whether Niraparib affected SRC kinase as a possible mechanism of inhibition of pSTAT3. We treated PDAC cell lines MIA PaCa-2 and PANC-1 and ovarian cancer cell lines OVCAR8 and PEO1 with multiple doses of Niraparib. We found that treatment with Niraparib for 24 hours or 36 hours (PEO1) diminished pSTAT3 activation and reduced phosphorylation of SRC at tyrosine 416 (pSRC) (**Figure 3A**).

Previous studies have shown that murine fibroblast 3T3 cells transfected with v-SRC undergo transformation by upregulating pSTAT3 (33). To further characterize the effects of Niraparib on SRC activation, we utilized wild-type murine 3T3 fibroblasts, which display minimal SRC kinase activity, and 3T3 cells, which overexpress constitutively active v-SRC (34–36). First, we examined the effect of Niraparib on pSTAT3 activation. We observed that Niraparib treatment of v-SRC transformed 3T3 cells resulted in decreased pSTAT3 levels (**Figure 3B**). Conversely, pSTAT3 levels were not significantly reduced in wild-type 3T3 cells, which exhibit minimal SRC kinase activity (34). We also tested the effect of Niraparib on pSRC activity. We found that Niraparib also reduced the levels of p-SRC in v-SRC 3T3 cells (**Figure 3C**). We then analyzed the cytotoxicity of Niraparib on these cell lines with different pSRC levels of activation. Our data show that v-SRC-overexpressing 3T3 cells were more sensitive to cytotoxic effects of Niraparib than wild-type 3T3 cells (**Figure 3D**). Our results suggest that inhibition of pSTAT3 by Niraparib is partially mediated through suppression of SRC activity.

To further assess whether Niraparib directly or indirectly inhibits SRC kinase in cells, we performed a cellular thermal shift assay (CETSA) (24), which is expected to detect protein-ligand interactions in a cellular context (**Figure S3A**). The decreased melting temperature (Tm) of cellular SRC in MIA PaCa-2 and OVCAR8 cells after 20 µM Niraparib treatment indicated that Niraparib bound endogenous SRC and destabilized it in these cells (**Figure 3E**). We next sought to discern whether the disturbance of thermal stability of SRC in cells is mediated through direct Niraparib binding or secondary cellular metabolites of Niraparib. To assess this, we conducted a cell-free *in vitro* thermal shift assay (25) using human recombinant SRC protein. The left shift of the melting curve of SRC protein in the presence of Niraparib compared to that of SRC incubated with DMSO suggests that a high concentration of Niraparib destabilizes SRC directly *in vitro* (**Figure 3F**), which is consistent with the results shown in CETSA (**Figure 3E**). Dasatinib, a clinical SRC tyrosine kinase inhibitor (37), shows the opposite thermal shift, indicating direct stabilization of SRC. These results suggest that Niraparib directly binds SRC at a binding site that may be distinct from that bound by Dasatinib.

In contrast, incubation with Olaparib at an equally high concentration did not result in an evident shift in the melting curve of SRC compared to DMSO control (**Figure S3**). Collectively, these data provide further evidence that Niraparib-mediated inhibition of the STAT3 pathway may be attributed to direct inhibition of SRC kinase activity.

### Niraparib treatment reduces phosphorylation and activation of STAT3 and SRC in patient primary tumor samples

**Figure 4:**
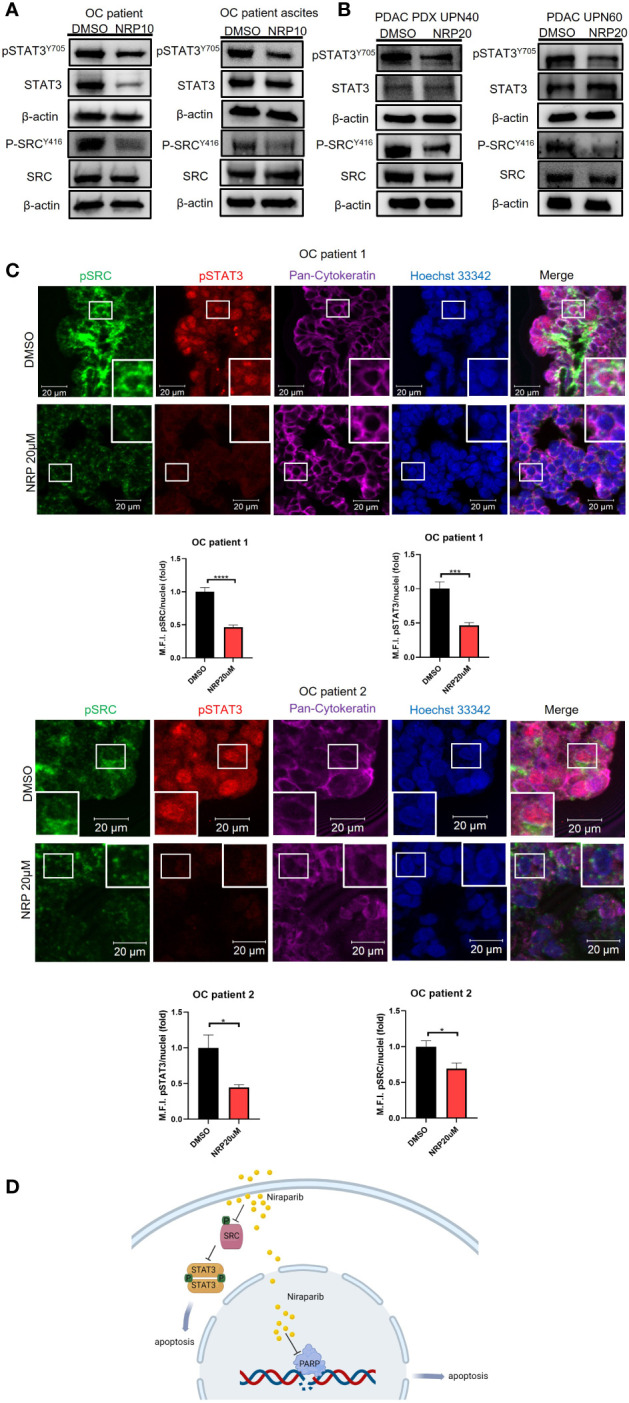
NRP treatment reduces pSTAT3 and pSRC in OvCa and PDAC patient tumor samples. **(A)** Western blot analysis of pSTAT3 and pSRC levels in OvCa patient primary tumor cells or ascites cells after 24h NRP treatment. β-actin served as a loading control. **(B)** Western blot analysis of levels of pSTAT3 and pSRC in PDAC-derived PDX tumor cells or PDAC patient primary tumor cells treated with NRP for 24h. β-actin served as a loading control. **(C)** Expression of pSTAT3 and pSRC in OvCa patient tumor slices treated with DMSO or NRP for 24h were examined by fluorescent immunohistochemistry and confocal microscopy. Representative images are shown from two OvCa patients. Red, pSTAT3; Green, pSRC; Magenta, pan-Cytokeratin; Blue/Hoechst 33342, nucleus. Cytokeratin-positive cell clusters demonstrate malignant tumor tissue. Scale bars = 20 μm. Histograms show quantification of M.F.I. of pSTAT3 and pSRC normalized to nuclear staining. Quantification was performed using ImageJ software, and at least five fields were quantified for each condition group. Data are presented as mean ± SEM. Unpaired two-tailed Student t-test, *p<0.05, ***p<0.001, ****p<0.001. **(D)** Niraparib induces tumor cell apoptosis through two mechanisms: Niraparib inhibits PARP, preventing DNA damage repairs in cells with BRCA mutations, thus causing tumor cell synthetic lethality. Our data show that Niraparib also interferes with SRC/STAT3 pathway to increase apoptosis of tumor cells with or without BRCA mutations.

To provide clinical relevance to our findings, we next investigated the effect of Niraparib on pSTAT3 and pSRC in ovarian and PDAC patients’ fresh tumor samples. Ovarian cancer patient tumor cells were collected from tumor tissues and patient ascites and subsequently incubated with Niraparib for 24 hours. Western blot analysis showed a significant reduction of pSTAT3 and pSRC in the samples incubated with Niraparib (**Figure 4A**). Furthermore, reduced pSTAT3 and pSRC levels after Niraparib treatment were also found in cells isolated from pancreatic cancer patient-derived xenograft tumors and directly from patient tumors (**Figure 4B**). In addition, ex vivo slices of ovarian cancer patient tumors were directly cultured as described (26) and treated with DMSO or 20 μM Niraparib for 24 hours. Immunofluorescent immunohistochemistry (IHC-IF) staining of pSTAT3 and pSRC on these tumor slices showed that STAT3 activity and SRC activity were dramatically downregulated by Niraparib treatment (**Figure 4C**). These data suggest that Niraparib inhibits STAT3 and SRC activities in fresh ovarian and PDAC patient tumor samples. In agreement with previous reports (38, 39) that indicate the importance of pSTAT3 and pSRC in PDAC, IHC-IF staining showed both elevated activated STAT3 and SRC in tumor cells of human PDAC cancer tissues (**Figure S4A**). Moreover, through data analysis from The Cancer Genome Atlas database, we found that higher SRC expression at the mRNA and protein levels is associated with worse PDAC patient survival (**Figure S4B**), further validating SRC as a promising therapeutic target for PDAC treatment.

In summary, our study suggests that Niraparib interferes with SRC/STAT3 pathway to increase apoptosis of tumor cells with or without BRCA mutations (**Figure 4D**).

## Discussion

Although PARPis were initially approved only for cancer patients with underlying defects in DNA damage repair, Niraparib has been recently approved as first-line maintenance therapy for advanced ovarian cancer in adults who responded to platinum-based chemotherapy, regardless of BRCA1/2 status (15). In line with these observations, we show that Niraparib efficiently inhibits ovarian and PDAC cancer cell growth regardless of BRCA1/2 mutations. Furthermore, Niraparib treatment exhibited higher tumor cell cytotoxicity than did Olaparib. Previous reports demonstrated higher PARP1 trapping ability and greater tumor exposure for Niraparib than other PARPis (18, 40), which may account for Niraparib’s better antitumor effects. However, these properties associated with Niraparib do not explain why it also effectively kills tumor cells with wild-type BRCA1/2. In our current study, we show that Niraparib downregulates STAT3 activity. We further show that expression of constitutively activated STAT3C significantly abrogates Niraparib-induced apoptosis in PDAC and ovarian cancer cells. It is widely documented that persistently active STAT3 facilitates tumor progression in human PDAC and ovarian cancers in part by inducing anti-apoptosis and pre-metastatic niche formation, thus serving as a potential therapeutic target (38, 41, 42). Given that we and others previously showed that STAT3 signaling also promotes the development of acquired resistance to various therapeutic agents in PDAC and ovarian cancer models, including PARPis (5, 43–45), our current findings suggest that the ability of Niraparib to inhibit pSTAT3 can cause increased apoptosis, providing a mechanism underlying Niraparib’s more substantial antitumor effects.

Despite sharing some molecular mechanisms of action, PARPis differ significantly among themselves in various parameters, such as binding affinities for the different PARP family members, protein expression regulation, and capacity to inhibit various intracellular kinases (17, 46, 47). Like our observations with STAT3, Niraparib, but not Olaparib or other PARPis, has been shown to non-canonically inhibit nucleotide salvage pathway rate-limiting deoxycytidine kinase, which may produce antagonistic effects when combined with nucleoside analogs (48). Furthermore, computational and *in-vitro* analyses of the unique polypharmacological kinase profiles of PARPis revealed that, in contrast to Olaparib, Niraparib could bind and inhibit several kinases involved in oncogenic signaling (47), suggesting that the unique capacity of Niraparib to inhibit STAT3 may be explained by secondary-target inhibition of upstream kinases.

Numerous reports showed that non-receptor tyrosine kinase SRC is involved in oncogenic STAT3 activation across various human cancers (39, 49). In line with these reports, our data show that Niraparib treatment downregulates Y416 phosphorylation of SRC in pancreatic and ovarian cancer cells and v-SRC-transformed murine fibroblasts. Of interest, our results show that only v-SRC transformed 3T3 cells, but not WT 3T3 cells, are susceptible to Niraparib-mediated STAT3 inhibition and cell proliferation. These findings are consistent with Niraparib exerting its antitumor effects through targeting the oncogenic SRC/STAT3 axis. Nevertheless, Antolin et al. (47) presented no biological function data on Niraparib’s effects on the kinases, and SRC kinase was not among the targets of Niraparib in their in-silico study. One likely explanation for the discrepancy is that our studies were all performed in cells treated with Niraparib. Although we have detected pSRC as a target of Niraparib, it remains to be determined whether Niraparib regulates other kinases activated in ovarian or pancreatic cancer cells to impact STAT3 activity and increase Niraparib’s antitumor effects.

Our work indicates that Niraparib destabilizes SRC directly, as shown by an *in vitro* thermal shift assay, whereas Dasatinib, an FDA-approved SRC inhibitor, stabilizes SRC, implying distinct protein-ligand interactions by Dasatinib and Niraparib. Dasatinib occupies the ATP binding pocket of SRC and thus inhibits enzymatic activity (50). However, the SRC binding site targeted by Niraparib remains to be elucidated but is potentially distinct from the ATP binding pocket. Presumably, the binding of Niraparib at a specific site elicits a SRC protein conformational change, leading to the destabilization of SRC protein in CETSA.

Finally, aberrantly activation of SRC occurs in approximately 70% of PDAC patients and contributes to tumorigenesis and progression of pancreatic cancer (39, 51, 52). Similarly, activated SRC is found in 36% of primary ovarian cancers, and inhibiting SRC can reduce tumor growth (49), suggesting that in addition to blocking DNA damage repair, targeting both STAT3 and SRC in tumor cells can be an advantage of Niraparib over Olaparib. Thus, our study provides a scientific rationale for using Niraparib not only in ovarian cancer but also in PDAC patients regardless of their BRCA1/2 status.

## Data availability statement

The raw data supporting the conclusions of this article will be made available by the authors, without undue reservation.

## Ethics statement

This study was reviewed and approved by IRB-approved protocols #18004, #19450, and #06129 at City of Hope. The patients/participants provided their written informed consent to participate in this study. The animal study was reviewed and approved by IACUC 10003.

## Author contributions

Study design, QZ, AK, AM, HY. Methodology, QZ, AK, Y-JL, AM, TA, CZ, NS, RB, XP, LM. Writing and editing, QZ, AK, Y-JL, AM, TA, NS, RB, LR-R, HY. Supervision, HY, LR-R. All authors contributed to the article and approved the submitted version.

## Funding

This study was supported by the Markel-Friedman Accelerator Fund at City of Hope and Grants from Rivkin Center for Ovarian Cancer and the Mary Kay Foundation.

## Acknowledgments

We thank the staff at Pathology-Solid Tumor Core, Analytical Cytometry Core, and Light Microscopy Core at the Beckman Research Institute, City of Hope, for their technical support and assistance. This study was supported by grants from Rivkin Center for Ovarian Cancer and the Mary Kay Foundation, and the Markel-Friedman Accelerator Fund at City of Hope. We would like to thank BioRender.com for enabling us to make schematic figure.

## Conflict of interest

The authors declare that the research was conducted in the absence of any commercial or financial relationships that could be construed as a potential conflict of interest.

## Publisher’s note

All claims expressed in this article are solely those of the authors and do not necessarily represent those of their affiliated organizations, or those of the publisher, the editors and the reviewers. Any product that may be evaluated in this article, or claim that may be made by its manufacturer, is not guaranteed or endorsed by the publisher.
