# Supplementary material for: Niraparib-induced STAT3 inhibition increases its antitumor effects
Source: Front Oncol. 2022 Oct 17;12:966492. doi: 10.3389/fonc.2022.966492 (PMC9618811; doi:10.3389/fonc.2022.966492)
Supplement: Supplementary file 2 [file DataSheet_1.pdf]

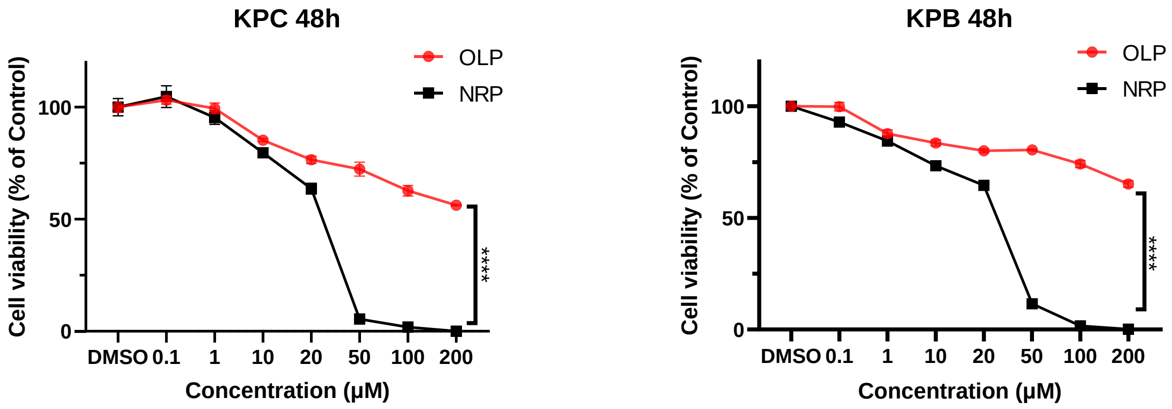

Figure S1. Cell viability assay confirmed superior antitumor effects of NRP to OLP in murine PDAC cancer cells with or without BRCA2 mutation. 5,000 cells were seeded in 96 well plates the day before treatment. Next day, cells were treated with indicated concentrations of OLP or NRP for 48h. DMSO concentration was 0.2%. Cell viability was determined by CellTiter-Glo assay following protocol. The data shown are representative of two independent experiments that were conducted in at least triplicate. Two-way ANOVA was performed. ****p<0.0001.

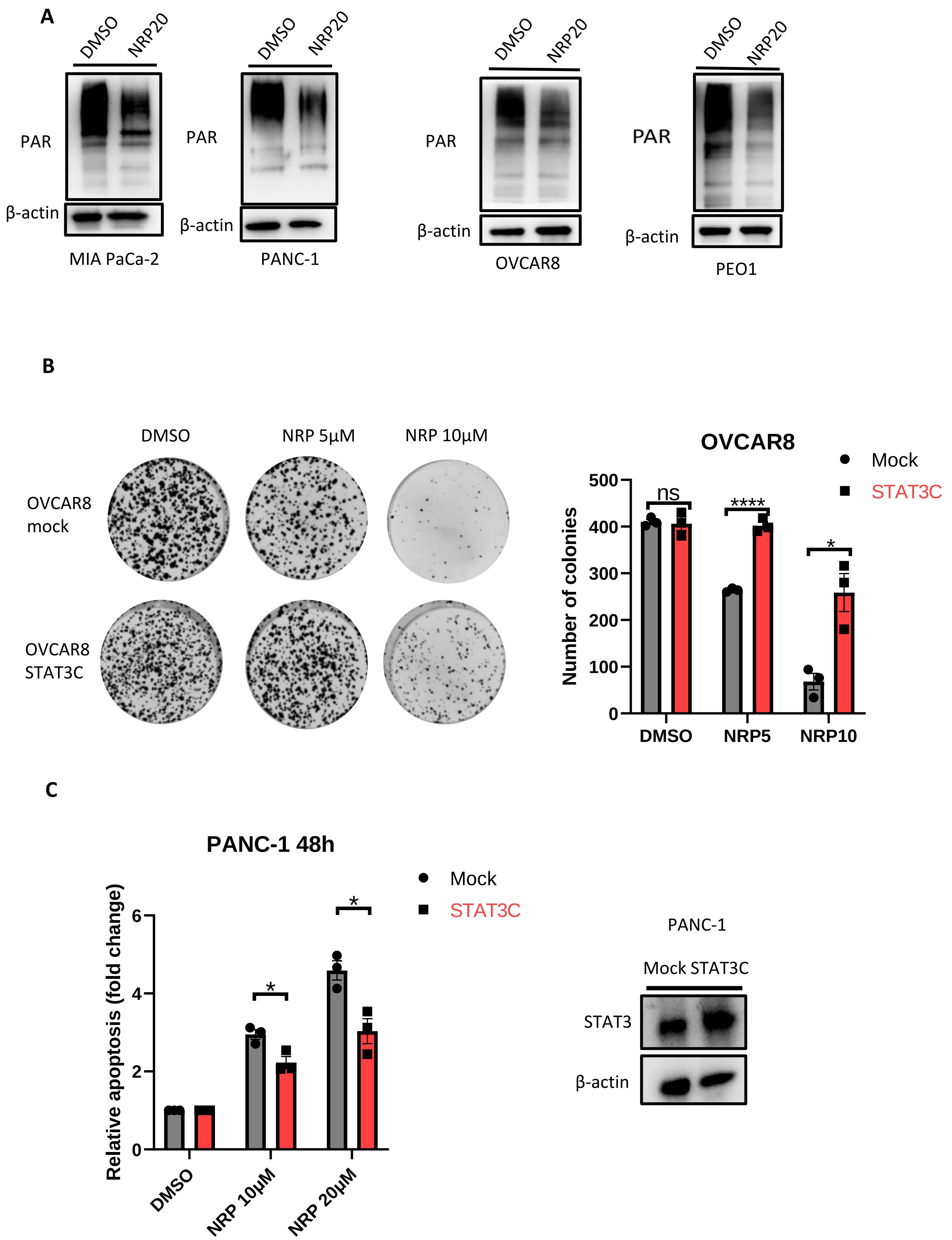
A
DMSO
NRP20
PAR
β-actin
MIA PaCa-2
PANC-1
OVCAR8
PEO1
B
DMSO
NRP 5µM
NRP 10µM
OVCAR8
mock
OVCAR8
STAT3C
OVCAR8
Mock
STAT3C
Number of colonies
0
100
200
300
400
500
ns
****
*
DMSO
NRP5
NRP10
C
PANC-1 48h
Mock
STAT3C
Relative apoptosis (fold change)
0
2
4
6
*
*
DMSO
NRP 10µM
NRP 20µM
PANC-1
Mock STAT3C
STAT3
β-actin

FigureS2. Overexpression of STAT3C rescues the apoptosis induced by Niraparib. **(A)** Human PDAC cell lines and OvCa cell lines were treated with NRP for 22h and followed by further 2h incubation with fresh NRP medium. Then H_2O_2 (final concentration 0.5 mM) was added and incubated for 15min. Cell lysates were collected and total parylation level of protein was detected by Western blotting. β-actin served as a loading control. **(B)** Overexpression of STAT3C restores the viability of cells treated with NRP assessed by colony formation assay. 3,000 OVCAR8 mock or STAT3C-overexpressing cells were seeded in 6-well plate and treated with DMSO, NRP 5μm or 10μm for 11 days. The representative images of three independent experiments are shown here with the quantification of colonies (right panel). Data are presented in mean± SEM (N=3). Unpaired two-tailed Student t-test, ns, not significant, *$p<0.05$, ****$p<0.0001$. **(C)** PANC-1 mock or STAT3C-overexpressing cells after 48h treatment with NRP were stained with Annexin V-APC and propidium iodine and analyzed by flow cytometry. Unpaired two-tailed Student t-test between mock and STAT3c-overexpressing cells at various treatments was conducted. Data are shown in mean± SEM (N=3). *$p<0.05$. Right panel showing increased ectopic STAT3c expression in STAT3c-overexpressing Panc1 cells by Western blotting. β-actin served as an internal control.

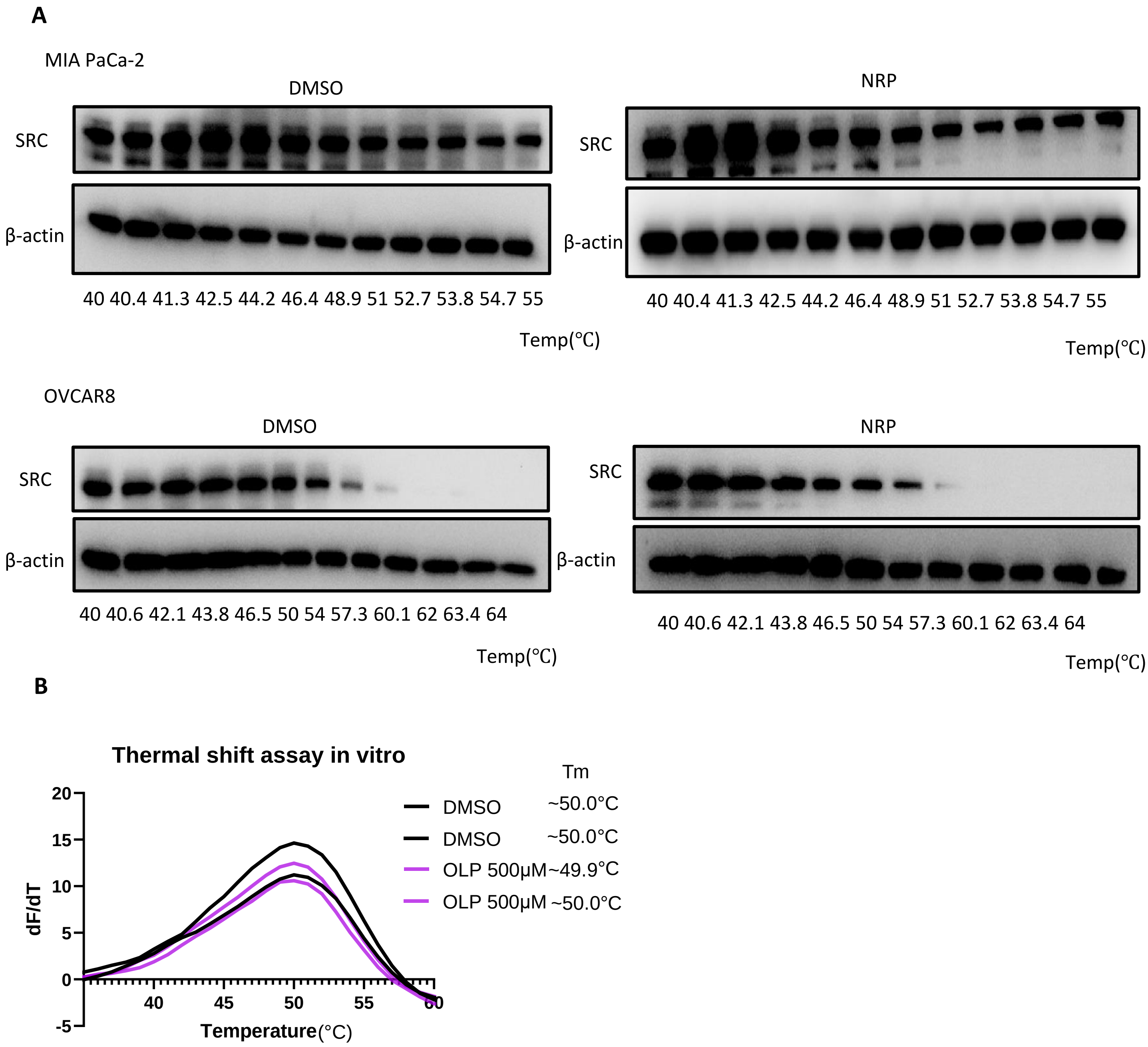

Figure3S. Niraparib interacts with SRC in MIA PaCa-2 and OVCAR8 cells. **(A)** In cell thermal shift assay was performed on MIA PaCa-2 and OVCAR8 cells treated with DMSO or 20μM NRP for 2h at 37℃. Endogenous SRC level was detected by Western blotting. β-actin served as an internal control. Three independent experiments were conducted. **(B)** *In vitro* thermal shift assay on human recombinant SRC protein indicated that Olaparib does not bind to SRC at high concentration. Data shown are the two SRC protein melting curves at distinct conditions.

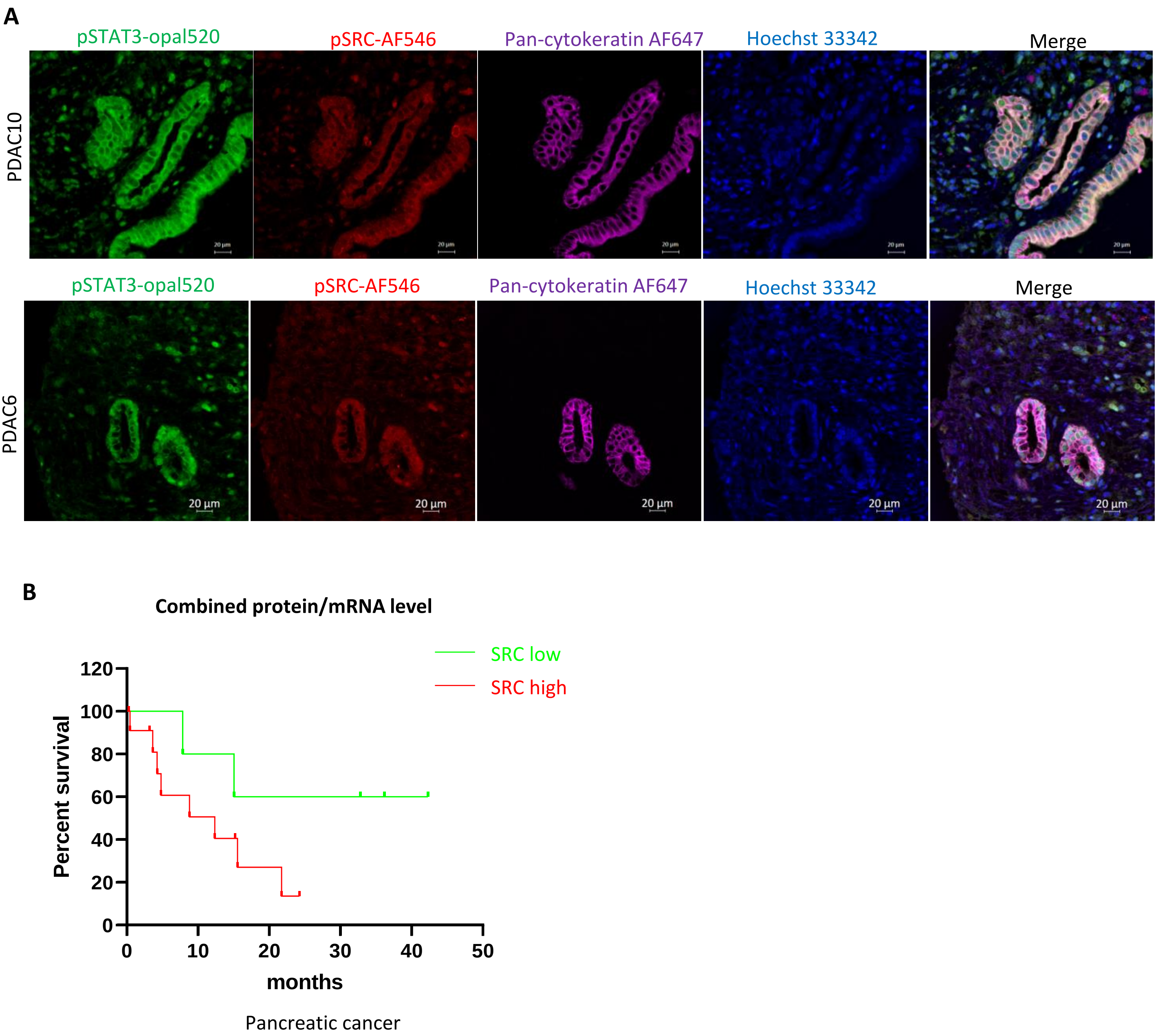

Figure 4S. p-SRC and p-STAT3 are correlated in human PDAC tumors. **(A)** p-SRC (Green) and p-STAT3 (Red) were colocalized in tumor cells on 2 human PDAC tumor tissues shown by fluorescent-IHC staining. Cytokeratin-positive cell clusters demonstrated tumor cells. Representative images are presented. Scale bars = 20 µm. **(B)** Survival analysis based on SRC mRNA and protein levels of pancreatic cancer patients from The Cancer Genomic Atlas (TCGA). High SRC expression indicates worse survival in pancreatic cancer patients.
